# Hwanhon Decoction Ameliorates Cognitive Impairment and Suppresses Neuroinflammation in a Chronic Cerebral Hypoperfusion Mouse Model: Involvement of Key Genes Identified by Network Pharmacology

**DOI:** 10.3390/genes16070746

**Published:** 2025-06-26

**Authors:** Sieun Kang, Chiyeon Lim, Sehyun Lim, Kyoung-Min Kim, Suin Cho

**Affiliations:** 1 Department of Korean Medicine, College of Korean Medicine, Dong-Eui University, Busan 47227, Republic of Korea; shilakang@naver.com; 2 Department of Radiology, Massachusetts General Hospital and Harvard Medical School, Charlestown, MA 02129, USA; rachun@hanmail.net (C.L.); favor119@daum.net (S.L.); 3 School of Public Health, Far East University, Eumseong 27601, Republic of Korea; 4 Department of Korean Medicine, School of Korean Medicine, Pusan National University, Yangsan 50612, Republic of Korea

**Keywords:** vascular dementia, chronic cerebral hypoperfusion, bilateral common carotid artery stenosis, cognitive impairment, neuroinflammation

## Abstract

Background: With an aging population, dementia prevalence is increasing in Korea. Vascular dementia (VaD), often caused by cerebrovascular disease (CVD), is more common in Korea compared to Western countries. Hwanhon decoction, a traditional medicine containing Ephedrae Herba, Armeniacae Semen, and Glycyrrhizae Radix et Rhizoma, is traditionally used for CVD-related loss of consciousness. This study aimed to assess the cognitive improvement and anti-inflammatory effects of Hwanhon decoction extract (HHex) in a mouse model of VaD caused by chronic cerebral hypoperfusion (CCH). Methods: Key pharmacologically active ingredients of Hwanhon decoction were identified using network pharmacology analysis. VaD was induced in C57Bl/6 male mice through bilateral common carotid artery stenosis (BCAS). Mice were divided into sham surgery, BCAS control, low-dose HHex (L-HHex), and high-dose HHex (H-HHex) groups (*n* = 5/group). After CCH induction, L-HHex or H-HHex was administered thrice weekly for six weeks. Cognitive function, inflammatory markers, and RNA sequencing data were analyzed. Results: HHex administration reduced cognitive impairment and mitigated CCH-induced astrocyte activation. Inflammatory responses mediated by reactive astrocytes were suppressed, and network pharmacology predicted central proteins influencing HHex’s activity. Conclusions: HHex alleviated cognitive dysfunction and reduced inflammation in a VaD mouse model, suggesting its potential as a therapeutic agent for vascular dementia associated with impaired cerebral blood flow.

## 1. Introduction

Dementia is a group of diseases that affect memory and other cognitive functions, causing abnormal behavioral and personality changes, loss of emotional function, and social or occupational dysfunction. Thus, dementia, rather than cancer or cardiovascular diseases, has been a more frequent cause of concern for older adults [1,2,3]. Patients with dementia are becoming more common in Korea, and their numbers are predicted to rise steadily, surpassing 1.3 and 3 million by 2030 and 2050, respectively [4]. Furthermore, 50–60% of patients with dementia in the United States have Alzheimer’s disease (AD), 15–20% have vascular dementia (VaD), and 15–20% have AD and VaD [2,5]. VaD causes approximately 15–20% of dementia cases in North America and Europe and approximately 30% in Asia and poorer nations. VaD, being a cerebrovascular disorder, can impair different central nervous system (CNS) regions. Similarly, VaD reportedly occurs in several patients with stroke [6]. As VaD progresses to severe symptoms, patients experience difficulties in multiple areas, including a reduction in living capacity, and their burden of care is reportedly greater than that of patients with AD [7,8].

Donepezil and galantamine are clinically used drugs specifically developed for treating AD [9,10]. Similarly, lecanemab and donanemab have recently been approved by the US Food and Drug Administration (FDA), raising patient expectations for treatment [11,12,13]. In contrast, no drug has been approved by the FDA for VaD treatment. Although donepezil has demonstrated therapeutic efficacy in mild cognitive impairment, its precise mechanism of action remains unclear [5,14]; hence, uncertainty persists regarding its potential effectiveness in treating severe cognitive impairment. Furthermore, VaD manifests at a younger age than AD does, which may cause a significantly longer treatment duration [2,14]. However, the lack of approved treatments for VaD may be attributed to the relatively low interest in this condition, stemming from its lower prevalence than that of AD. Thus, considering the symptom severity and illness duration, studies on VaD, particularly in Asian countries, are necessary.

In AD, abnormal protein accumulation causes brain cell death, prompting treatment strategies that increase neurotransmitter availability or prevent neuronal degeneration [12,15,16]. VaD is a disease in which the brain tissue is damaged owing to cerebrovascular disease (CVD), and the current primary VaD treatment is to control vascular risk factors [9]. Hence, investigations into therapeutic agents for VaD treatment are necessary, and future VaD medications may operate on several targets at once, exerting beneficial effects on pathological processes, including inflammation, apoptosis, and cell adhesion [17]. Therefore, the potential contributions of traditional Asian medicines should be considered.

The Hwanhon decoction (Huanhun-Tang) is a traditional medicine prescription described in the Synopsis of Prescriptions of the Golden Chamber (*Jinguiyaolue*), written by *Zhang Zhongjing*, a famous physician during the Eastern Han Dynasty of China. This decoction has been used for emergency purposes. It comprises three medicinal herbs, namely ephedra, apricot kernels, and licorice. Recently, it has been used more frequently in clinical practice because it is effective in treating insomnia caused by cardiovascular disease [18,19]. Similarly, Hwanhon decoction has been recorded in the *Donguibogam*, a classical medical text published in Korea, and its composition is similar to that in the Synopsis of Prescriptions of the Golden Chamber. It has traditionally been used to treat febrile diseases and, recently, cognitive dysfunction. However, evidence regarding its efficacy is insufficient; hence, it was used in this study.

We sought to confirm whether the target protein predictions made using network pharmacology analysis were consistent with the results of RNA sequencing analysis because herbal medicines contain abundant ingredients, making it challenging to determine which specific ingredient exerts therapeutic effects. Moreover, a single component cannot exhibit only one activity. To elucidate the effects of the Hwanhon decoction in a chronic cerebral hypoperfusion (CCH)-induced VaD mouse model, we conducted the Y-maze test, novel object recognition test (NORT), and elevated plus maze (EPM) test to evaluate CCH-induced cognitive dysfunction. In addition, brain tissues were observed using immunofluorescence staining to confirm the degree of activation of immune cells involved in the inflammatory brain damage caused by ischemia and the immune response suppression activity of the Hwanhon decoction. RNA sequencing was performed to identify the genes and proteins involved in the regulation of the inflammatory response.

## 2. Materials and Methods

### 2.1. Network Pharmacology Analysis

The Traditional Chinese Medicine Systems Pharmacology Database and Analysis Platform (TCMSP), which is primarily used in Chinese medicine publications involving numerous network pharmacology study techniques, is a representative database for network pharmacology studies frequently conducted based on Asian traditional medicine databases [20]. The TCMSP database was used for the analysis in this study. After selecting the active ingredients, pharmacokinetically active substances with potential potency were chosen based on absorption, distribution, metabolism, and excretion (ADME) properties. Examples of ADME variables include oral bioavailability (OB), which indicates the ability of an orally administered drug to be delivered to the body; drug-likeness (DL), which is used to estimate the pharmacokinetic optimality of a compound and helps identify potential lead compounds; and Caco-2 permeability, which is used to assess drug absorption in the gastrointestinal tract. As recommended by the TCMSP, the cutoff values for each variable were set as follows: OB ≥ 20%, DL ≥ 0.18, and Caco-2 ≥ 0 [21,22].

### 2.2. Animals and Experimental Design

Male C57BL/6 mice (6-week-old; weight, 20–22 g) were purchased from Samtako Bio (Osan, Gyeonggi-do, Republic of Korea) and housed in polypropylene cages in a breeding room with a 12 h light/dark cycle and a temperature of 24 ± 4 °C. The mice were allowed free access to standard feed and water and were acclimated to the enclosed environment for ≥1 week before the experiment. All experimental procedures used in this study, including surgery, were approved by the Ethics Committee of Pusan National University (approval number PNU 2023-0259).

### 2.3. VaD Modeling in Mice

To mimic VaD using CCH, we performed a bilateral common carotid artery stenosis (BCAS) operation. Briefly, isoflurane in N_2_O/O_2_ (70%/30%) gas was used to anesthetize the mice until they did not react to mechanical tail stimulation. Following a midline incision in the neck area, the common carotid artery (CCA) was isolated from the surrounding tissue. Subsequently, the CCA was wrapped with a micro-coil (0.18 mm inner diameter) to induce BCAS. Throughout the entire surgical process, the body temperature was maintained at 37 ± 0.5 °C using a device equipped with a temperature sensor attached to the rectum.

### 2.4. Reagents

Physiological saline was obtained from Joongwae Pharmaceutical (Seoul, Republic of Korea); the tissue fixative (optimal cutting temperature, OCT) compound for frozen sections was purchased from Bio Basic (Markham, ON, Canada), and the physiological buffer (phosphate buffered saline, PBS) was obtained from Thermo Fisher Scientific (Waltham, MA, USA).

### 2.5. Preparation of Aqueous Extract of Hwanhon Decoction (HHex)

The medicinal ingredients of Hwanhon decoction corresponding to the daily dose for an adult weighing 60 kg are listed in Table 1. The Hwanhon decoction formula was soaked in boiling water for 2 h; residues were removed using non-woven fabric to obtain 270 mL of HHex. Because this solution is equivalent to a daily dose for a 60 kg human adult, it can be converted to a dosage of 4.5 mL/kg body weight. Considering the metabolic rate in mice, this would be approximately 13 times the human dosage [23]; hence, 58.5 mL/kg of body weight would correspond to this. Therefore, we set the low-dose HHex (L-HHex) and high-dose HHex (H-HHex) to 10 and 50 mL/kg, respectively. The daily dose was orally administered to mice at once because administering the medicine to lab animals thrice daily would be stressful. When orally administered to the mice, L-HHex was diluted five-fold with distilled water to adjust the dose volume to that of H-HHex.

### 2.6. Mouse Treatment with HHex

**Figure 1:**
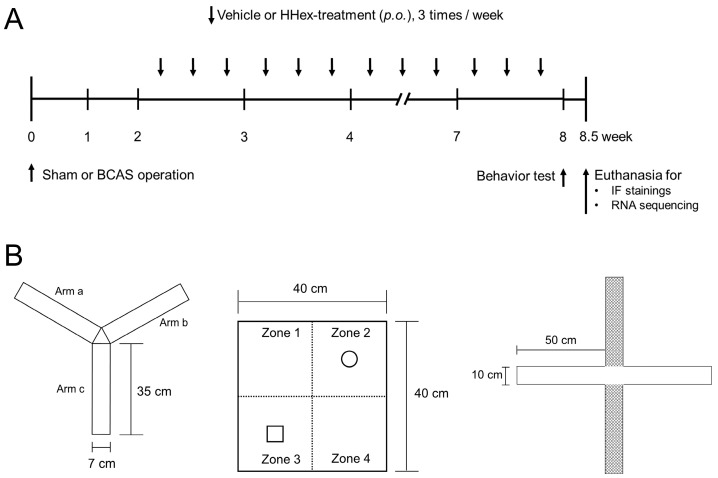
Overview of the experimental procedure and specification of instruments for measuring behavior. (**A**) Vehicle or Hwanhon decoction (HHex) were treated thrice weekly for 6 weeks following bilateral common carotid artery stenosis (BCAS) operation. Each group contained five mice. (**B**) Behavior tests, such as the Y-maze test, the novel object recognition test (NORT), and the elevated plus maze (EPM) test, were conducted 8 weeks following BCAS surgery. The individuals underwent anesthesia 8.5 weeks after BCAS surgery to harvest their brains for RNA sequencing and immunofluorescence (IF) staining.

The mice were randomly divided into four experimental groups (*n* = 5 each), namely sham operation, BCAS control, BCAS + L-HHex, and BCAS + H-HHex groups. In the sham group, the surgical procedures, except for BCAS, were applied during the surgical procedure, and 50 mL/kg of primary distilled water was administered rather than HHex during the HHex administration period. Similarly, the BCAS control group was administered 50 mL/kg of primary distilled water, and the L-HHex and H-HHex groups were administered the corresponding HHex thrice weekly throughout the HHex administration period (Figure 1A).

### 2.7. Measuring Behavioral Changes Caused by BCAS-Induced CCH

The short-term spatial working memory of the mice was evaluated using the Y-maze test. A mouse with intact working memory, which includes intact prefrontal cortex functioning, will recall the arms it has visited in the past and exhibit a propensity to enter the less visited arm [24]. The mice were placed in a maze that resembled the letter Y and had three arms of the same shape (Figure 1B). Moreover, the mice were allowed to explore the maze for a certain period before they were examined. Each experimental animal was placed in the maze center and allowed to roam freely for 10 min. A digital camcorder was used to record and evaluate mouse motion in the maze to determine how thoroughly they successively investigated each of the three arms; the degree to which the three arms were studied sequentially was expressed as a percentage of the total number of times they were explored (for instance, C–A–B, B–C–A and A–B–C). The following formula was used to determine the spontaneous alternation behavior: % alternation = ((number of alternations)/(total number of arm entries − 2)) × 100. For the Y-maze test, mice were allowed to acclimate to the maze for 2 days (5 min daily) before measurement.

NORTs are used to assess changes in cognitive function using rodent traits that make them more interested in new items than in familiar ones [25,26]. Two pre-adaptation trials were conducted once daily, 2 days before the NORT. Each mouse was given 5 min to explore an open-field arena (40 × 40 × 40 cm) without objects to facilitate field box adaptation (Figure 1B). An object adaptation period was provided on the cognitive function assessment day. Briefly, two identical objects were positioned in opposite corners (zones 2 and 3) of the open-field arena (Figure 1B), and the mice were given 10 min to investigate the two items to allow familiarization. After 30 min, a familiar (F) object from the acclimation trial was swapped for a novel (N) object shortly before the experimental mouse was returned to the open-field arena for measurement. They were allowed to freely navigate the objects in the arena for 10 min, and a digital camcorder was used to capture their behavior. For each experimental group, object search time and discrimination ratio (DR) analyses were conducted using the formula: total N time/(N time + F time) × 100.

Anxiety-like behavior is commonly measured using the EPM tests. The mouse innate dislike of open, high places and their innate curiosity in unfamiliar settings served as the foundation for the EPM test. All arms were made available to the mice, and they were free to move about them. Indicators of open space-induced anxiety in mice include the number of animals that enter the open arms and the amount of time they spend there [26,27]. Thus, increased duration and transition between closed arms indicate anti-anxiety behavior. Because the maze was designed to make experimental animals feel anxious, a closed arm with a 45 cm-high wall, 50 cm above the laboratory floor, crossed an open passage horizontally (Figure 1B). Each experimental animal was positioned in the middle of the cross and allowed to roam freely along open or closed arms for 5 min. The mice were allowed to explore the maze for 2 days (5 min daily) before measurement to facilitate acclimation in the EPM. After each mouse was observed in the maze or arena, the maze or arena was cleaned with 70% alcohol to prevent the next experimental mouse from being attracted by the scent of other individuals. The behavioral pathways captured using a digital camera were examined for movement patterns in the maze using SMART software (version 3.0.06, PanLab, Barcelona, Spain).

### 2.8. Variations in Body Weight, Blood Pressure (BP), and Heart Rate (HR)

The body weight, BP, and HR of the experimental mice were measured at 2-week intervals during the experimental period to determine whether the reduction in blood flow and drug administration affected these parameters. Non-invasive BP and HR were measured using a specialized device (Visitech System, BP-2000, Apex, NC, USA) attached to the mouse tail.

### 2.9. Assessment of the Blood Serum Electrolytes

Whole blood was drawn under deep anesthesia and spun at 1500× *g* for 15 min at 4 °C to extract serum. Blood serum electrolytes were examined because drastic changes in electrolytes can also affect behavior. To track and rule out possible electrolyte imbalances, serum concentrations of electrolytes, such as potassium (K^+^), chloride (Cl^−^), and sodium (Na^+^), were measured using an electrolyte analyzer (Dri-Chem 3500i, Fuji, Japan).

### 2.10. Animal Euthanasia, Cardiac Perfusion, and Brain Tissue Preparation via Frozen Section

The experimental mouse abdomen was cut at the center, and PBS was used to perform cardiac perfusion. A 21-gauge needle was used to puncture the left ventricle and block the pulmonary artery, and the needle was secured in the ascending aorta. The right atrium was sliced with scissors immediately after perfusion to remove the perfused blood from the body. Fixation was performed through perfusion with 4% paraformaldehyde once perfusion with PBS was sufficiently advanced. Mouse brains were successively fixed in 10%, 20%, and 30% sucrose solutions for cryoprotection. Subsequently, the tissue was frozen in OCT compound and refrigerated at −80 °C. Each mouse brain slice was deposited on a 25 μm-thick slide glass using a cryostat (Leica, Wetzlar, Germany) and refrigerated at −80 °C until needed.

### 2.11. Brain Tissue Immunofluorescence Staining

Brain tissue sections were removed from the refrigerator and allowed to dry. After 1 h of reaction at 25 °C with blocking buffer (5% bovine serum albumin), the blocking buffer was cleaned, and diluted primary antibodies of cluster of differentiation 68 (CD68+), ionized calcium-binding adaptor molecule 1, neuronal nuclear protein, and glial fibrillary acidic protein (GFAP) (#97778, #17198, #94403, and #12389, respectively; Cell Signaling Technology, Danvers, MA, USA) were added and reacted for 12 h at 4 °C. After the primary antibodies were washed thrice with PBS for 5 min, the tissue section was exposed to a diluted secondary antibody (Abcam, Cambridge, UK) for 2 h at 25 °C for fluorescence production. A glass slide fixation solution (ab104139; Abcam, Cambridge, UK) containing 4,6-diamidino-2-phenylindole was dropped over the tissue, and this was covered with a cover slide; the edges were sealed with nail polish after the secondary antibody was washed thrice with PBS for 5 min each. A fluorescence microscope (Ni-U, Nikon, Tokyo, Japan) was used for tissue observation, and ImageJ software (version 1.54g, NIH, MD, USA) was used to combine the tissue photographs.

### 2.12. Brain Tissue RNA Sequencing Analysis

After behavioral analysis, the mice were euthanized using CO_2_. The brain tissue was extracted under cold experimental conditions, and the cerebral cortex, which included the white matter (WM), was separated and rapidly frozen. Total RNA was isolated using TRIzol reagent, and only RNA with a confirmed amount and purity ratio suitable for analysis was used in this study. The total RNA quality was assessed using capillary electrophoresis (Agilent 2100 Bioanalyzer, Agilent, Santa Clara, CA, USA). After isolating mRNA from total RNA to synthesize cDNA, high-throughput sequencing was conducted. The RNA expression levels were determined by comparing the expression levels of the control and experimental groups to those of the sham group, with the sham group expression level set as the baseline. The relative expression comparison was displayed as a heat map using the MeV program (https://mev.tm4.org, accessed on 1 December 2023). After selecting proteins for analysis, protein–protein interaction (PPI) networks were generated using the STRING database (https://string-db.org/, accessed on 1 December 2023) and visualized using the network analysis software Cytoscape (version 3.8.2).

### 2.13. Statistical Analysis

The results are presented as mean ± standard deviation (SD). Prior to hypothesis testing, the distribution of each variable was assessed by the Shapiro–Wilk test implemented in SigmaPlot 12.0 (Systat Software Inc., Chicago, IL, USA). Variables with a normal distribution (Shapiro–Wilk *p* > 0.05) were compared among groups by one-way analysis of variance (ANOVA), followed by Holm–Sidak *post hoc* tests. Variables that deviated from normality (Shapiro–Wilk *p* < 0.05) were analyzed by the Kruskal–Wallis test with Dunn’s multiple-comparison post hoc analysis. A *p*-value < 0.05 was considered statistically significant.

## 3. Results

### 3.1. Network Pharmacology Analysis for HHex

**Figure 2:**
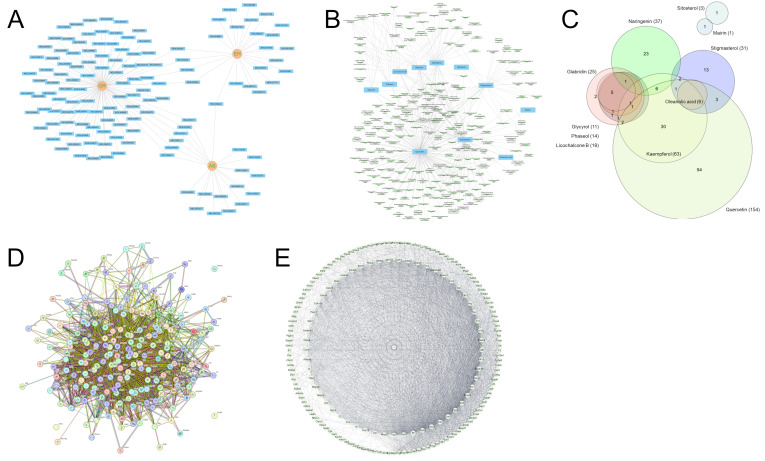
Screening of the active target proteins in HHex components using the TCMSP database. (**A**) The three medicinal herbs (Glycyrrhizae Radix et Rhizoma (GR), Ephedrae Herba (EH), and Armeniacae Semen (AS)) that constitute HHex and their active ingredients were selected, and a network of 11 overlapping ingredients and proteins predicted to be regulated by them was constructed (**B**,**C**). A protein–protein interaction (PPI) network of these target proteins was constructed using the STRING database (**D**), and the frequency of interaction with other proteins was visualized (**E**).

When ADME variables were applied through the TCMSP database, 24 ingredients were selected from Ephedrae Herba (EH), 20 from Armeniacae Semen (AS), and 102 from Glycyrrhizae Radix et Rhizoma (GR) (Supplementary Tables S1–S3). After excluding the duplicates, 135 components were selected (Figure 2A). Among the 135 ingredients, 11 overlapping ingredients were tested for target proteins and were entered into the TCMSP database, yielding 228 targets (Figure 2B). Ingredients such as quercetin, kaempferol, and naringenin were confirmed to have more targets than others (Figure 2B). Among the 11 common ingredients, 10, excluding stigmasterol, were present in GR (Figure 2A; Tables S1 and S3), suggesting that GR may have diverse and relatively strong activities compared with those of other medicinal herbs. By constructing a PPI network using the STRING database for the 228 targets selected as described above, a network similar to that in Figure 2D was obtained, and this was reconstructed using the Cytoscape program to obtain a network similar to that in Figure 2E. In Figure 2E, the number of lines connecting each target indicates the number of interacting proteins; hence, the inner circle has a relatively higher frequency of interaction than that of the outer circle. Therefore, proteins such as tumor necrosis factor, AKT serine/threonine kinase 1, and interleukin-6 can be predicted to be primarily regulated by HHex (Figure 2E). However, whether the targets predicted through the network pharmacology analysis are regulated by HHex administration is unknown.

### 3.2. Effects of BCAS Operation and HHex Treatment on Physiological Parameters in Mice

**Figure 3:**
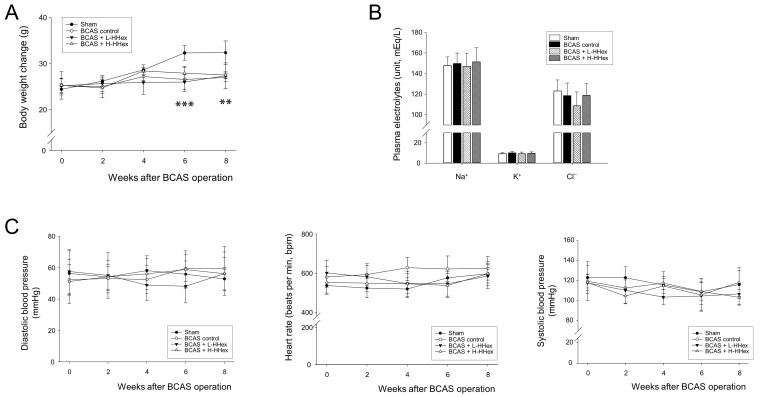
Influence of BCAS-induced CCH in mice and the effect of HHex treatment on physiological parameters. (**A**) The sham group steadily gained weight; however, the groups that underwent BCAS surgery showed a reduction in weight gain. BCAS surgery or HHex administration did not affect serum electrolytes (**B**), blood pressure, or pulse rate (**C**). Data are expressed as mean ± standard error (*n* = 5). ** *p* < 0.01, *** *p* < 0.001 BCAS control group vs. sham group. CCH: chronic cerebral hypoperfusion.

A significant reduction in body weight was observed from week 6 in the BCAS-operated group, whereas no change in body weight was noted in the HHex-treated groups compared with the BCAS control group (Figure 3A). There were no physiological changes in the blood serum electrolyte content, as CCH-induced BP, HR changes, and HHex administration had no effect (Figure 3B,C). Therefore, although the BCAS operation caused weight loss, the other physiological parameters were not affected by surgery or HHex administration.

### 3.3. Behavioral Disorder Assessment Using the Y-Maze Test, NORT, and EPM Test

**Figure 4:**
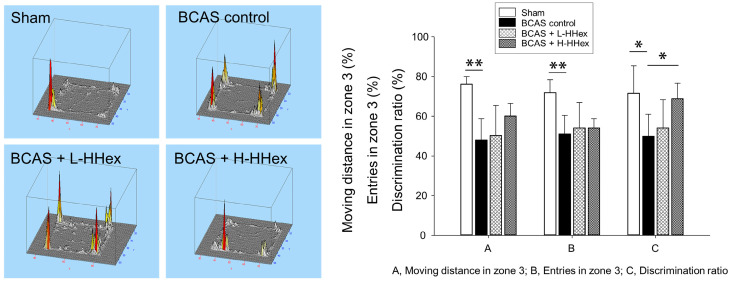
NORT using an open-field arena. Mice that underwent BCAS surgery showed a decrease in activity and the ability to recognize new objects in the open field. Although HHex did not affect the activity, H-HHex suppressed the decrease in the ability to recognize novel objects. Data are expressed as mean ± standard error (*n* = 5). * *p* < 0.05, ** *p* < 0.01 vs. compared group.

**Figure 5:**
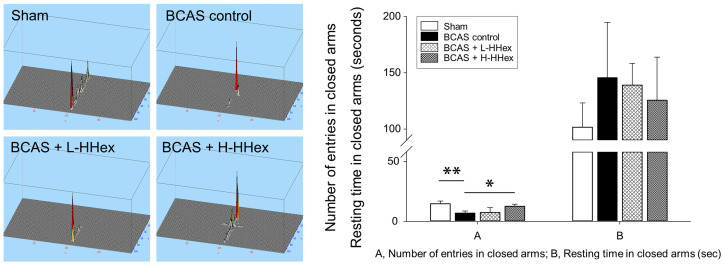
Results of the EPM test. Mice that underwent BCAS surgery exhibited reduced exploration of the closed arm in the EPM; however, H-HHex treatment significantly increased the exploration of the closed arm compared with that in the BCAS control group. No differences were observed in the time spent in the closed arm between each experimental group. Data are expressed as mean ± standard error (*n* = 5). * *p* < 0.05, ** *p* < 0.01 vs. compared group.

The Y-maze test is a behavioral experiment used to evaluate an animal’s spatial memory ability [24]. It comprised a Y-shaped maze (Figure 1B), and memory ability was evaluated by observing the animals’ process of navigating the maze. No significant differences were observed in the first-arm choice latency, triple-arm alternation, or total arm entries in the Y-maze among the experimental groups, including the sham group, and the SD was large overall (Figure S1). In the NORT, the natural curiosity of rodents for novel objects over familiar objects is leveraged to evaluate changes in cognitive function [25,26]. In the BCAS control group, a significant decrease was observed in the moving distance in the arena, number of entries in zone 3, and DR compared with that in the sham group, and a significant suppression of the decrease in DR was confirmed in the H-HHex-administered group (Figure 4). The EPM test is a behavioral test designed to study anxiety in rodents [26,27]. It comprises a platform with four arms, two open and two closed, elevated above the ground (Figure 1B). The test is based on the premise that rodents naturally fear open and elevated spaces; therefore, increased time spent and transitions between the closed arms indicate anti-anxiety behavior. Although the resting time in the closed arms did not differ among the experimental groups, the number of entries into the closed arms by the experimental mice was significantly reduced by BCAS-induced CCH, and this reduction was notably mitigated by H-HHex administration (Figure 5). Therefore, this study confirmed that maintaining CCH through BCAS operation for 8 weeks caused significant cognitive dysfunction in experimental mice and that administering a relatively high concentration of HHex (H-HHex) at intervals of approximately 2 days for 6 weeks suppressed this decline in cognitive function.

### 3.4. Identification of Cellular Markers Involved in the Inflammatory Response in the Brain

**Figure 6:**
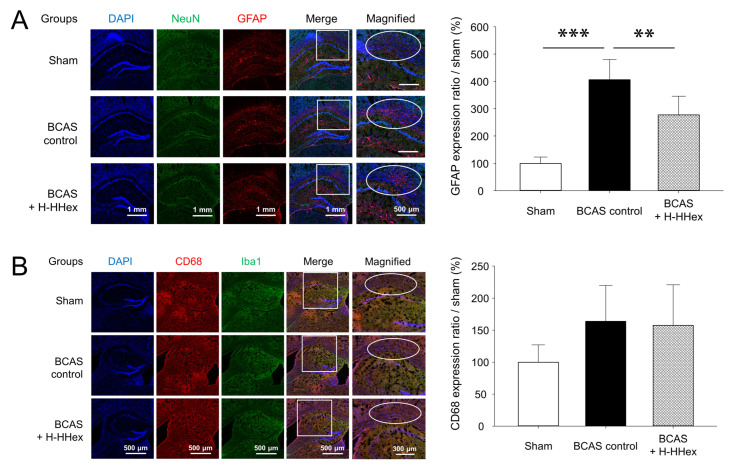
Observation of expression of GFAP-positive astrocytes and CD68-positive microglia using IF staining. (**A**) GFAP expression in the cerebral cortex, including the corpus callosum, significantly increased after BCAS-induced CCH, and this increase in expression was inhibited by HHex treatment. (**B**) BCAS-induced CCH or HHex treatment did not affect CD68 expression levels. Data are expressed as mean ± standard error (*n* = 5). ** *p* < 0.01, *** *p* < 0.001 vs. compared group.

Astrocytes and microglia are glial cells that play crucial roles in neuroinflammation, an essential factor in chronic CCH-induced VaD [28,29]. WM damage is a common VaD feature that causes cognitive decline, particularly in older adults [30,31,32]. In this study, we aimed to determine whether CCH upregulates the expression of these two cell types and whether H-HHex administration modulates the expression of inflammatory cells in the WM region. The number of GFAP-positive astrocytes around the cerebral cortex, particularly in the WM, was increased; this increase was suppressed by H-HHex administration (Figure 6A). However, the number of CD68-positive microglia did not significantly increase in the BCAS control group compared with that in the sham group, and H-HHex administration did not affect the number of microglia (Figure 6B).

### 3.5. RNA Sequencing Analysis and PPI Network Construction

**Figure 7:**
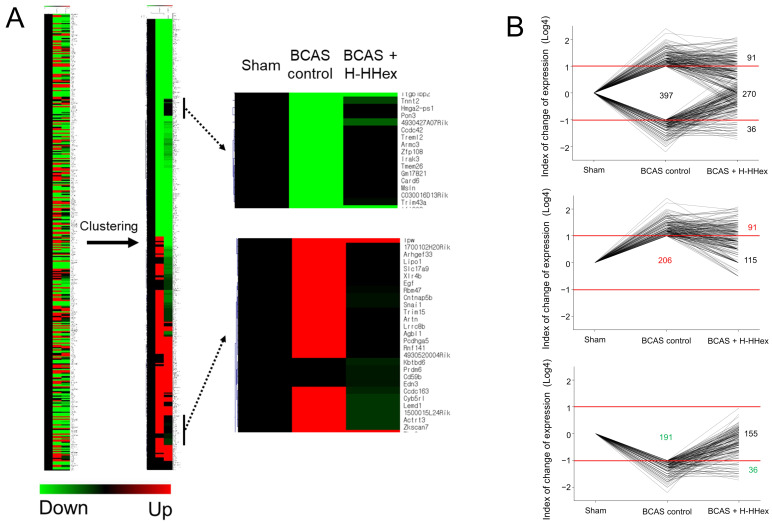
Analysis of gene expression using RNA sequencing. (**A**) Clustering analysis of the gene expression changes owing to BCAS-induced CCH and HHex administration reveal distinct expression patterns. Normalized data were hierarchically clustered according to the gene expression ratio. The intensity of the green or red color indicates levels of downregulation or upregulation in gene expression, respectively. (**B**) CCH-altered genes were categorized based on their direction of change (up-regulated or down-regulated), and the effect of HHex on the expression of these genes was visualized.

The gene expression level was confirmed through RNA sequencing analysis, and the changes in expression are shown in Figure 7. Gene expression levels in the BCAS control group were considered significant and selected as the analysis target if they decreased by <1/4 or increased by ≥4-fold compared with those in the sham group (Figure 7A). Clustering was performed using log transformation to understand the increase or decrease in values. As shown in the horizontal bar at the bottom of Figure 7A, the green area represents genes with decreased expression compared with that in the sham group, and the red area represents genes with increased expression. Therefore, genes showing significant decreases (green area) or increases (red area) in expression owing to CCH can be considered related to the pathological changes in the experimental mice. Hence, genes regulated by H-HHex administration can be found in the black marks on the rightmost panel in Figure 7A. To determine how CCH and H-HHex administration affect gene expression, we visualized the number of genes that showed increased or decreased expression (Figure 7B). Thus, 397 and 206 genes showed a >4-fold increase and a <1/4 decrease, respectively, owing to CCH. Of the 206 genes with increased expression, 115 were identified; of the 191 genes with decreased expression, 155 exhibited expression levels within a 4-fold range (>1/4 and <4), indicating near-normal expression patterns. Overall, 68% of the genes were regulated, and the effect of H-HHex administration on genes with decreased expression owing to CCH was more evident. Tables S4 and S5 detail the genes whose expression was altered—either up- or down-regulated—by CCH operation and subsequently modulated by H-HHex administration.

**Figure 8:**
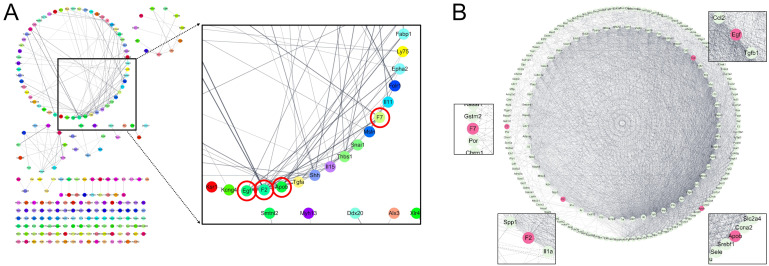
PPI network constructed using the STRING database. (**A**) A PPI network was constructed targeting genes regulated by HHex, and proteins with a high frequency of interaction with other proteins were selected. The string between the proteins indicates known or predicted interactions. (**B**) Comparison with the target proteins predicted through network pharmacology analysis confirmed that proteins predicted through network pharmacology analysis, such as Egf, F2, Apob, and F7, were regulated by HHex treatment and played crucial roles in the PPI network.

The STRING database was used to construct a PPI network targeting genes up-regulated or down-regulated by H-HHex administration to identify proteins that play a central role. The results were visualized using Cytoscape, and proteins such as epidermal growth factor (Egf), prothrombin (coagulation factor II, F2), and apolipoprotein B (Apob) frequently interacted with other proteins (Figure 8A). The proteins identified through RNA sequencing to exert a significant effect were compared with those predicted to be affected by HHex through network pharmacology analysis. The interaction frequencies with other proteins differed. However, proteins such as Egf, F2, Apob, and coagulation factor VII (F7) showed the same results as predicted (Figure 8B). GO enrichment of H-HHex-responsive genes (Tables S6–S8) revealed that H-HHex down-regulated CCH-induced DNA-replication terms (e.g., DNA duplex unwinding; Table S6) while rescuing axonogenesis-related processes (Table S7). Across all responsive transcripts, oxidoreductase activity and RNA-pol II transcription factor functions were prominently enriched (Table S8), underlining H-HHex’s dual impact on genomic stability, metabolic homeostasis, and neuronal repair.

## 4. Discussion

Dementia is a disorder in which an individual loses independence in everyday functions owing to cognitive impairment; this loss represents a significant departure from the previous level of performance [33]. In 2020, the number of patients with dementia among the South Korean population aged ≥65 was estimated to be approximately 840,000, with a prevalence rate of approximately 10.3%. Similarly, the number of patients with dementia is increasing in Asia and is expected to triple by 2050. This is related to risk factors such as aging, obesity, smoking, and hypertension. Among the various types of dementia, VaD is a dementia type caused by CVD and is considered a significant health challenge in Asia. The number of patients with dementia in South Korea increases as the population ages. VaD is the second most common type of dementia after AD, accounting for approximately 15–20% of patients with dementia [2,4,5].

VaD and AD have been believed to have completely different roots; however, a recent study has shown that they share a common vascular component and that these vascular factors can interact and coexist rather than being mutually exclusive [34]. Because the incidence of CVD and heart disease increases with age, AD is usually accompanied by stroke or vascular rupture. CVDs contribute to the progression and development of AD, causing the frequent co-occurrence of VaD and AD. VaD is estimated to account for 20% of all dementia cases. However, this percentage is expected to be significantly higher in Asian countries. Recent Korean studies have reported that VaD accounts for 20–30% of all dementia cases in Korea [35,36,37].

VaD treatment involves careful management of the risk factors for CVD and the use of antithrombotic drugs to prevent stroke recurrence while enhancing cognitive function. Medications such as aspirin and warfarin are used to prevent CVD recurrence and progression, whereas other drugs are used to control risk factors such as hyperlipidemia, high blood pressure, diabetes, and atrial fibrillation. Acetylcholinesterase inhibitors such as donepezil, rivastigmine, and memantine are used to improve cognitive function. Cholinesterase inhibitors also help improve cognitive dysfunction in patients with VaD [14,36,38]. Although no FDA-approved treatments exist for VaD, cholinesterase inhibitors such as donepezil, galantamine, and rivastigmine, and N-methyl-D-aspartate (NMDA) receptor antagonists such as memantine, which are occasionally used to treat AD, are primarily administered because some patients with VaD also show AD symptoms. However, cholinesterase inhibitors have side effects such as nausea, diarrhea, and insomnia, and NMDA receptor antagonists have side effects such as headache and dizziness; therefore, safe and effective treatments for VaD need to be developed [11,12,13,14,36,38].

HHex is a traditional medicine prescription comprising EH, AS, and GR. Zuo et al. [39] found that EH extract alleviated blood–brain barrier disruption and cerebral edema after subarachnoid hemorrhage in rats. Kao et al. [40] revealed that GR has neuroprotective effects and improves behavioral performance. Therefore, we aimed to confirm whether the pharmacological activity of Hwanhon decoction was clearly observed by conducting a preclinical study on mice, as Hwanhon decoction has been frequently used in treating cognitive impairment in Korean medicine clinical practice; however, evidence regarding its effectiveness remains lacking.

To simulate VaD in humans, we wrapped micro-coils around both common carotid arteries to induce CCH. Eight weeks after BCAS, mice exhibited significant impairments in object recognition (NORT) and increases in anxiety-like behavior (EPM), both of which were significantly attenuated by H-HHex administration (Figure 4 and Figure 5). In contrast, Y-maze measures of spatial working memory—including first-arm choice latency, spontaneous alternation rate, and total arm entries—did not differ significantly among sham, BCAS control, and H-HHex groups (Figure S1). Thus, while H-HHex appears to preserve recognition memory and reduce anxiety-like responses under CCH, we did not observe a clear enhancement in spatial working memory in the current cohort. Future studies with larger sample sizes and dedicated spatial-memory paradigms (e.g., Morris water maze) are required to determine whether HHex can improve Y-maze performance under CCH. The ability to recognize unfamiliar objects and reduce depressive tendencies was clearly observed by conducting the NORT (Figure 4) and the EPM test (Figure 5). This effect was believed to be owing to the suppression of astrocyte activation and reduction in immune responses by HHex (Figure 6A), which reduced degenerative changes in the brain WM. Network pharmacology analysis was performed on the medicinal ingredients that constitute the HHex, the material of this study, to select potentially active compounds.

A PPI network was constructed for the proteins predicted to be regulated by these ingredients, and proteins predicted to play a central role when HHex is active were selected (Figure 2). In addition, RNA sequencing analysis was performed by extracting the cerebral cortex, including the mouse WM, to identify genes regulated by H-HHex (Figure 7); an interaction network of proteins predicted to be regulated by these genes was constructed (Figure 8A). As described above, the major proteins predicted using network pharmacology analysis were compared with those showing expression changes identified through RNA sequencing analysis. Proteins such as Egf, F2, and Apob that played the most essential role in the extract activity were predicted through network pharmacology analysis (Figure 8B). Furthermore, approximately 68% of the genes that showed altered expression owing to ischemia were regulated through H-HHex administration (Figure 7B).

Egf is found in neurons, astrocytes, oligodendrocytes, and microglia of the CNS, and its signaling can affect inflammatory responses in the skin and CNS. Moreover, Egf signaling contributes to neuroinflammation in CNS disorders, and Egf can reappear in reactive astrocytes after an insult, which can be neuroprotective; however, Egf can also contribute to neurotoxicity [41]. F2 is a bloodborne protein that induces neuroinflammation, leading to neurodegeneration and cognitive decline. Similarly, F2 can activate microglia, which release pro-inflammatory cytokines that damage neurons [42,43]. Apob may contribute to compromised blood–brain barrier integrity, which can cause neuroinflammation [44,45,46]. F7 interacts with inflammation and the coagulation system, which can contribute to neuroinflammation [47,48,49]. Thus, Egf, F2, Apob, and F7 are proteins involved in neuroinflammation, the activation of neuroinflammatory cells such as microglia and astrocytes, and in the inflammatory response owing to CCH and subsequent cognitive dysfunction.

When the CNS is damaged, such as through stroke, dementia, or brain injury, astrocytes are transformed into reactive astrocytes, such as in hypertrophy and hyperplasia, and GFAP is used to label these reactive astrocytes [50,51,52]. BCAS-induced CCH increased the expression of astrocytes in the cerebral cortex, particularly in the corpus callosum; this overexpression was suppressed through H-HHex administration (Figure 6A). In addition, although not statistically significant (Figure 6B), CD68, a well-known microglial marker, tended to be increased by CCH, suggesting that BCAS-induced CCH increases the activation of astrocytes and microglial cells in the corpus callosum region.

The HHex sample used in this study comprised three medicinal herbs, which is relatively few compared with the number of medicinal herbs in other traditional medicine prescriptions; however, elucidating the specific mechanism of action is difficult when a prescription contains many ingredients. In this study, among the numerous ingredients in the medicinal herbs that constitute this prescription, 11 overlapping ingredients were selected through network pharmacology analysis to identify target proteins predicted to be regulated by these ingredients (Figure 2). Furthermore, HHex administration was confirmed to suppress the decrease in cognitive function induced by CCH in mouse brain (Figure 4 and Figure 5). Similarly, proteins involved in suppressing the decrease in cognitive function after HHex administration were identified using RNA sequencing analysis (Figure 7 and Figure 8). Common proteins were identified between the proteins predicted through network pharmacology analysis and those with expression regulated using HHex administration, particularly Egd, F2, and Apob, which play the most central roles (Figure 8). The activity of various components in the Hwanhon decoction may be owing to complex interactions and may depend on the activity of several major ingredients. We primarily focused on selecting major ingredients and targets and identifying target proteins involved in the activity and did not explore the major mechanisms of action. In a follow-up study, we aim to use quercetin and naringenin, which have many targets among the individual components selected in this study, to determine whether HHex inhibitory activity on cognitive function decline depends on these components. Additionally, we intend to elucidate the molecular biological mechanisms underlying HHex treatment, including the expression of Egf, F2, and Apob, which are regulated by HHex.

## 5. Conclusions

This study was conducted to determine whether Hwanhon decoction can suppress cognitive dysfunction caused by BCAS-induced CCH in mice. In summary, HHex administration mitigates certain cognitive deficits induced by BCAS-mediated CCH—specifically, it preserves object recognition and alleviates anxiety-like behaviors—yet it does not significantly enhance spatial working memory as assessed by the Y-maze in our present study. Network pharmacology and transcriptomic analyses have highlighted key protein targets underlying HHex’s effects. Follow-up work with larger cohorts and complementary spatial memory assays will be essential to fully establish HHex’s therapeutic potential in VaD models. This study provides a foundation for the effective application of Hwanhon decoction in addressing cognitive function decline resulting from cerebral hypoperfusion.

## Figures and Tables

**Table 1:** Medicinal ingredients that constitute Hwanhon decoction: herbal names, scientific names, and daily doses for humans (60 kg body weight).

Herbal Name	Scientific Name	Weight (g)
Ephedrae Herba	*Ephedra Sinica* Stapf	18
Armeniacae Semen	*Prunus armeniaca* L. var. *ansu* Maxim.	18
Glycyrrhizae Radix et Rhizoma	*Glycyrrhiza uralensis* Fischer	6
Total Amount		42

## Data Availability

The data that support the findings of this study are not publicly available due to ethical reasons but are available from the corresponding author (SC) upon reasonable request.

## Supplementary Materials

The following supporting information can be downloaded at: https://www.mdpi.com/article/10.3390/genes16070746/s1, Table S1: Active ingredients of Ephedrae Herba; Table S2: Active ingredients of Armeniacae Semen; Table S3: Active ingredients of Glycyrrhizae Radix et Rhizoma; Table S4: Genes underexpressed by CCH and up-regulated by HHex administration; Table S5: Genes overexpressed by CCH and down-regulated by HHex administration; Table S6: Analysis of biological process using GO terms; Table S7: Analysis of cellular components using GO terms; Table S8: Analysis of molecular function GO terms; Figure S1: Results of the Y-maze behavioral test.

## Abbreviations

The following abbreviations are used in this manuscript:

AD	Alzheimer’s disease
ADME	Absorption, distribution, metabolism, and excretion
AS	Armeniacae Semen
BCAS	Bilateral common carotid artery stenosis
CCH	Chronic cerebral hypoperfusion
CD68	Cluster of differentiation 68
CVD	Cerebrovascular diseases
DAPI	4,6-diamidino-2-phenylindole
DL	Drug likeness
DR	Discrimination ratio
EH	Ephedrae Herba
EPM	Elevated plus maze
GFAP	Glial fibrillary acidic protein
GR	Glycyrrhizae Radix et Rhizoma
HHex	Hwanhon decoction aqueous extract
H-HHex	High-dose HHex
L-HHex	Low-dose HHex
NMDA	N-methyl-D-aspartate
NORT	Novel object recognition test
OB	Oral bioavailability
OCT	Optimal cutting temperature
PPI network	Protein–protein interaction network
VaD	Vascular dementia
WM	White matter
