# Supplementary material for: Hwanhon Decoction Ameliorates Cognitive Impairment and Suppresses Neuroinflammation in a Chronic Cerebral Hypoperfusion Mouse Model: Involvement of Key Genes Identified by Network Pharmacology
Source: Genes (Basel). 2025 Jun 26;16(7):746. doi: 10.3390/genes16070746 (PMC12295633; doi:10.3390/genes16070746)
Supplement: Supplementary file 1 [file genes-16-00746-s001.zip › genes-3666545-supplementary.pdf]

Hwanhon Decoction Ameliorates Cognitive Impairment and Suppresses Neuroinflammation in a Chronic Cerebral Hypoperfusion Mouse Model: Involvement of Key Genes Identified by Network Pharmacology

List of supplementary Tables

Supplementary Table 1 (Table S1). Active ingredients of Ephedrae Herba.

Table S2. Active ingredients of Armeniacae Semen.

Table S3. Active ingredients of Glycyrrhizae Radix et Rhizoma.

Table S4. Genes underexpressed by CCH and up-regulated by HHex administration.

Table S5. Genes overexpressed by CCH and down-regulated by HHex administration.

Table S6. Analysis of biological process using GO terms.

Table S7. Analysis of cellular components using GO terms.

Table S8. Analysis of molecular function GO terms.

List of supplementary Figure

Figure S1. Results of the Y-maze behavioral test.

Table S1. Active ingredients of Ephedrae Herba.

Mol ID	Molecule name	MW	OB (%)	Caco-2	DL
MOL002083	Tricin	330.31	27.86	0.51	0.34
MOL002823	Herbacetin	302.25	36.07	0.12	0.27
MOL000422	Kaempferol	286.25	41.88	0.26	0.24
MOL000008	Apigenin	270.25	23.06	0.43	0.21
MOL000098	Quercetin	302.25	46.43	0.05	0.28
MOL000006	Luteolin	286.25	36.16	0.19	0.25
MOL000263	Oleanolic acid	456.78	29.02	0.59	0.76
MOL000358	Beta-sitosterol	414.79	36.91	1.32	0.75
MOL000449	Stigmasterol	412.77	43.83	1.44	0.76
MOL001494	Mandenol	308.56	42	1.46	0.19
MOL001506	Supraene	410.8	33.55	2.08	0.42
MOL001755	24-Ethylcholest-4-en-3-one	412.77	36.08	1.46	0.76
MOL001771	Poriferast-5-en-3beta-ol	414.79	36.91	1.45	0.75
MOL002881	Diosmetin	300.28	31.14	0.46	0.27
MOL004328	Naringenin	272.27	59.29	0.28	0.21
MOL005043	Campest-5-en-3beta-ol	400.76	37.58	1.32	0.71
MOL005190	Eriodictyol	288.27	71.79	0.17	0.24
MOL005573	Genkwanin	284.28	37.13	0.63	0.24
MOL005734	Eupatilin	344.34	29.39	0.75	0.38
MOL005842	Pectolinarigenin	314.31	41.17	0.7	0.3
MOL006775	Epi-Afzelechin	274.29	23.74	0.19	0.21
MOL008422	Afzelechin	274.29	28.54	0.06	0.21
MOL010546	ST069309	285.32	24.02	0.65	0.38
MOL011319	Truflex OBP	334.5	43.74	0.9	0.24

Table S2. Active ingredients of Armeniacae Semen.

Mol ID	Molecule name	MW	OB (%)	Caco-2	DL
MOL010921	Estrone	270.4	53.56	1.01	0.32
MOL010922	Diisooctyl succinate	342.58	31.62	0.72	0.23
MOL010923	11-docosenoic acid	338.64	28.56	1.21	0.26
MOL002211	11,14-eicosadienoic acid	308.56	39.99	1.22	0.2
MOL002372	(6Z,10E,14E,18E)-2,6,10,15,19,23-hexamethyltetracosa-2,6,10,14,18,22-hexaene	410.8	33.55	2.07	0.42
MOL000359	Sitosterol	414.79	36.91	1.32	0.75
MOL000449	Stigmasterol	412.77	43.83	1.44	0.76
MOL005030	Gondoic acid	310.58	30.7	1.2	0.2
MOL000953	CLR	386.73	37.87	1.43	0.68
MOL000211	Mairin	456.78	55.38	0.73	0.78
MOL002311	Glycyrol	366.39	90.78	0.71	0.67
MOL002332	STOCK1N-53032	297.38	27.79	0.84	0.33
MOL003410	Ziziphin_qt	472.78	66.95	0.49	0.62
MOL004355	Spinasterol	412.77	42.98	1.44	0.76
MOL004841	Licochalcone B	286.3	76.76	0.47	0.19
MOL004908	Glabridin	324.4	53.25	0.97	0.47
MOL005017	Phaseol	336.36	78.77	0.76	0.58
MOL005386	Vulgarin	264.35	29.21	0.03	0.2
MOL007207	Machiline	285.37	79.64	0.78	0.24
MOL012922	l-SPD	327.41	87.35	0.76	0.54

Table S3. Active ingredients of Glycyrrhizae Radix et Rhizoma.

Mol ID	Molecule name	MW	OB (%)	Caco-2	DL
MOL001484	Inermine	284.28	75.18	0.89	0.54
MOL001792	DFV	256.27	32.76	0.51	0.18
MOL000211	Mairin	456.78	55.38	0.73	0.78
MOL002311	Glycyrol	366.39	90.78	0.71	0.67
MOL000239	Jaranol	314.31	50.83	0.61	0.29
MOL002565	Medicarpin	270.3	49.22	1	0.34
MOL000263	Oleanolic acid	456.78	29.02	0.59	0.76
MOL000354	Isorhamnetin	316.28	49.6	0.31	0.31
MOL000359	Sitosterol	414.79	36.91	1.32	0.75
MOL003656	Lupiwighteone	338.38	51.64	0.68	0.37
MOL003896	7-Methoxy-2-methyl isoflavone	266.31	42.56	1.16	0.2
MOL000392	Formononetin	268.28	69.67	0.78	0.21
MOL000417	Calycosin	284.28	47.75	0.52	0.24
MOL000422	Kaempferol	286.25	41.88	0.26	0.24
MOL004328	Naringenin	272.27	59.29	0.28	0.21
MOL000445	8-Prenylwighteone	406.51	23.22	0.93	0.54
MOL000467	Castanin	298.31	23.54	0.77	0.27
MOL004804	18beta-glycyrrhetinic acid	470.76	22.05	0.1	0.74
MOL004805	(2S)-2-[4-hydroxy-3-(3-methylbut-2-enyl)phenyl]-8,8-dimethyl-2,3-dihydropyrano[2,3-f]chromen-4-one	390.51	31.79	1	0.72
MOL004806	Euchrenone	406.56	30.29	1.09	0.57
MOL004808	Glyasperin B	370.43	65.22	0.47	0.44
MOL004810	Glyasperin F	354.38	75.84	0.43	0.54
MOL004811	Glyasperin C	356.45	45.56	0.71	0.4
MOL004812	Glyasperins D	370.48	29.91	0.89	0.43
MOL004814	Isotrifoliol	298.26	31.94	0.53	0.42
MOL004815	(E)-1-(2,4-dihydroxyphenyl)-3-(2,2-dimethylchromen-6-yl)prop-2-en-1-one	322.38	39.62	0.66	0.35
MOL004820	Kanzonols W	336.36	50.48	0.63	0.52
MOL004823	Licoagropin	320.46	27.14	1.63	0.51
MOL004824	(2S)-6-(2,4-dihydroxyphenyl)-2-(2-hydroxypropan-2-yl)-4-methoxy-2,3-dihydrofuro[3,2-g]chromen-7-one	384.41	60.25	0	0.63
MOL004827	Semilicoisoflavone B	352.36	48.78	0.45	0.55
MOL004828	Glepidotin A	338.38	44.72	0.79	0.35
MOL004829	Glepidotin B	340.4	64.46	0.46	0.34
MOL004833	Phaseolinisoflavan	324.4	32.01	1.01	0.45

MOL004835	Glypallichalcone	284.33	61.6	0.76	0.19
MOL004838	8-(6-hydroxy-2-benzofuranyl)-2,2-dimethyl-5-chromenol	308.35	58.44	1	0.38
MOL004841	Licochalcone B	286.3	76.76	0.47	0.19
MOL004846	Apioglycyrrhizin_qt	470.76	23.73	0.1	0.74
MOL004848	Licochalcone G	354.43	49.25	0.64	0.32
MOL004849	3-(2,4-dihydroxyphenyl)-8-(1,1-dimethylprop-2-enyl)-7-hydroxy-5-methoxy-coumarin	368.41	59.62	0.4	0.43
MOL004855	Licoricone	382.44	63.58	0.53	0.47
MOL004856	Gancaonin A	352.41	51.08	0.8	0.4
MOL004857	Gancaonin B	368.41	48.79	0.58	0.45
MOL004863	3-(3,4-dihydroxyphenyl)-5,7-dihydroxy-8-(3-methylbut-2-enyl)chromone	354.38	66.37	0.52	0.41
MOL004864	5,7-dihydroxy-3-(4-methoxyphenyl)-8-(3-methylbut-2-enyl)chromone	352.41	30.49	0.9	0.41
MOL004866	2-(3,4-dihydroxyphenyl)-5,7-dihydroxy-6-(3-methylbut-2-enyl)chromone	354.38	44.15	0.48	0.41
MOL004878	Glycycoumarin	368.41	23.56	0.52	0.44
MOL004879	Glycyrin	382.44	52.61	0.59	0.47
MOL004882	Licocoumarone	340.4	33.21	0.84	0.36
MOL004883	Licoisoflavone	354.38	41.61	0.37	0.42
MOL004884	Licoisoflavone B	352.36	38.93	0.46	0.55
MOL004885	Licoisoflavanone	354.38	52.47	0.39	0.54
MOL004889	Licorice-saponin F3_qt	454.76	27.53	0.74	0.64
MOL004891	Shinpterocarpin	322.38	80.3	1.1	0.73
MOL004895	Licorice-saponin H2_qt	470.76	22.91	0.01	0.74
MOL004897	Licorice-saponin J2_qt	472.78	28.3	0.04	0.74
MOL004898	(E)-3-[3,4-dihydroxy-5-(3-methylbut-2-enyl)phenyl]-1-(2,4-dihydroxyphenyl)prop-2-en-1-one	340.4	46.27	0.41	0.31
MOL004901	Licorice-saponin K2_qt	470.76	27.79	0.05	0.75
MOL004904	Licopyranocoumarin	384.41	80.36	0.13	0.65
MOL004906	Hispaglabridin B	390.51	22.94	1.18	0.88
MOL004907	Glyzaglabrin	298.26	61.07	0.34	0.35
MOL004908	Glabridin	324.4	53.25	0.97	0.47
MOL004910	Glabranin	324.4	52.9	0.97	0.31
MOL004911	Glabrene	322.38	46.27	0.99	0.44
MOL004912	Glabrone	336.36	52.51	0.59	0.5
MOL004913	1,3-dihydroxy-9-methoxy-6-benzofurano[3,2-c]chromenone	298.26	48.14	0.48	0.43
MOL004914	1,3-dihydroxy-8,9-dimethoxy-6-benzofurano[3,2-c]chromenone	328.29	62.9	0.4	0.53

MOL004915	Eurycarpin A	338.38	43.28	0.43	0.37
MOL004935	Sigmoidin-B	356.4	34.88	0.42	0.41
MOL004941	(2R)-7-hydroxy-2-(4-hydroxyphenyl)chroman-4-one	256.27	71.12	0.41	0.18
MOL004945	(2S)-7-hydroxy-2-(4-hydroxyphenyl)-8-(3-methylbut-2-enyl)chroman-4-one	324.4	36.57	0.72	0.32
MOL004948	Isoglycyrol	366.39	44.7	0.91	0.84
MOL004949	Isolicoflavonol	354.38	45.17	0.54	0.42
MOL004950	Isoglycycoumarin	368.41	22.09	0.55	0.6
MOL004957	HMO	268.28	38.37	0.79	0.21
MOL004959	1-Methoxyphaseollidin	354.43	69.98	1.01	0.64
MOL004961	Quercetin der.	330.31	46.45	0.39	0.33
MOL004966	3'-Hydroxy-4'-O-Methylglabridin	354.43	43.71	1	0.57
MOL000497	Licochalcone a	338.43	40.79	0.82	0.29
MOL004974	3'-Methoxyglabridin	354.43	46.16	0.94	0.57
MOL004976	Daidzein dimethyl ether	282.31	24.29	0.98	0.24
MOL004978	2-[(3R)-8,8-dimethyl-3,4-dihydro-2H-pyrano[6,5-f]chromen-3-yl]-5-methoxyphenol	338.43	36.21	1.12	0.52
MOL004980	Inflacoumarin A	322.38	39.71	0.73	0.33
MOL004985	Icos-5-enoic acid	310.58	30.7	1.22	0.2
MOL004988	Kanzonol F	420.54	32.47	1.18	0.89
MOL004989	6-prenylated eriodictyol	356.4	39.22	0.4	0.41
MOL004990	7,2',4'-trihydroxy - 5-methoxy-3 - arylcoumarin	300.28	83.71	0.24	0.27
MOL004991	7-Acetoxy-2-methylisoflavone	294.32	38.92	0.74	0.26
MOL004993	8-prenylated eriodictyol	356.4	53.79	0.43	0.4
MOL004996	Gadelaidic acid	310.58	30.7	1.2	0.2
MOL000500	Vestitol	272.32	74.66	0.86	0.21
MOL005000	Gancaonin G	352.41	60.44	0.78	0.39
MOL005001	Gancaonin H	420.49	50.1	0.6	0.78
MOL005003	Licoagrocarpin	338.43	58.81	1.23	0.58
MOL005004	Gancaonin I	354.43	21.9	0.93	0.39
MOL005007	Glyasperins M	368.41	72.67	0.49	0.59
MOL005011	Kanzonol Z	406.51	21.77	0.5	0.76
MOL005012	Licoagroisoflavone	336.36	57.28	0.71	0.49
MOL005016	Odoratin	314.31	49.95	0.42	0.3
MOL005017	Phaseol	336.36	78.77	0.76	0.58
MOL005018	Xambioona	388.49	54.85	1.09	0.87
MOL005020	Dehydroglyasperins C	340.4	53.82	0.68	0.37
MOL000098	Quercetin	302.25	46.43	0.05	0.28

Table S4. Genes underexpressed by CCH and up-regulated by HHex administration.

Gene symbol	Sham	Control	HHT
Tnnt2	0.00	-2.20	0.73
Ifi203	0.00	-2.00	-0.10
Pon3	0.00	-2.00	0.00
Ifi27l2a	0.00	-1.93	-0.23
Mcm6	0.00	-1.89	0.14
Clec5a	0.00	-1.85	0.44
E130201H02Rik	0.00	-1.79	-0.79
Drc7	0.00	-1.79	-0.79
Rfx2	0.00	-1.79	-0.29
Fam179a	0.00	-1.79	-0.13
Hmga2-ps1	0.00	-1.79	0.00
Exoc3l4	0.00	-1.73	-0.07
C130060K24Rik	0.00	-1.73	0.22
Apol8	0.00	-1.66	-0.66
Olfr856-ps1	0.00	-1.66	-0.37
Dsn1	0.00	-1.66	-0.26
Itpripl1	0.00	-1.62	-0.62
Hfe2	0.00	-1.58	-0.42
Tbx15	0.00	-1.58	-0.42
Slc16a14	0.00	-1.58	-0.29
Hmmr	0.00	-1.58	-0.29
Ncaph	0.00	-1.58	0.14
6720468P15Rik	0.00	-1.50	-0.71
Itgb1bp2	0.00	-1.50	-0.71
Tmem114	0.00	-1.50	-0.34
Enpep	0.00	-1.50	-0.34
Cdc45	0.00	-1.50	0.23
Aaed1	0.00	-1.40	-0.90
Tmem8c	0.00	-1.40	-0.61
Slc27a5	0.00	-1.40	-0.61
F7	0.00	-1.40	-0.61
Procr	0.00	-1.40	-0.24
Nhlh1	0.00	-1.40	-0.11
Spc24	0.00	-1.40	0.07
Ang	0.00	-1.40	0.18
Egfl6	0.00	-1.38	-0.31
A230057D06Rik	0.00	-1.38	-0.06
5830418P13Rik	0.00	-1.35	-0.12
Trim65	0.00	-1.35	0.05
Gm11696	0.00	-1.29	-0.92
Scarna13	0.00	-1.29	-0.79
a	0.00	-1.29	-0.79
Epha2	0.00	-1.29	-0.79
Klf14	0.00	-1.29	-0.63

Tspan18	0.00	-1.29	-0.55
Dscc1	0.00	-1.29	-0.50
H2-Ob	0.00	-1.29	-0.50
Tcf15	0.00	-1.29	-0.50
Papolb	0.00	-1.29	-0.50
Zfp109	0.00	-1.29	-0.50
A330074K22Rik	0.00	-1.29	-0.50
Snora26	0.00	-1.29	-0.29
Sdk2	0.00	-1.29	-0.13
Tex11	0.00	-1.29	-0.13
Trpc1	0.00	-1.29	0.04
Bves	0.00	-1.29	0.11
Ptger2	0.00	-1.29	0.11
Nhs	0.00	-1.29	0.11
Pmfbp1	0.00	-1.29	0.21
Abhd15	0.00	-1.29	0.37
F2rl2	0.00	-1.25	-0.75
Xist	0.00	-1.25	-0.31
Spry4	0.00	-1.24	-0.30
Gm11747	0.00	-1.23	-0.44
A330033J07Rik	0.00	-1.23	-0.23
Shmt1	0.00	-1.23	-0.07
Armc3	0.00	-1.23	0.00
Zfp108	0.00	-1.23	0.00
Zfp119b	0.00	-1.23	0.06
Tusc5	0.00	-1.21	-0.42
Ddx20	0.00	-1.21	-0.34
Sp6	0.00	-1.16	-0.87
Tmprss6	0.00	-1.16	-0.66
Zan	0.00	-1.16	-0.66
Dlec1	0.00	-1.16	-0.66
Anxa1	0.00	-1.16	-0.50
Hist1h2bq	0.00	-1.16	-0.37
Mroh8	0.00	-1.16	-0.37
Pabpc4l	0.00	-1.16	-0.37
Folr2	0.00	-1.16	-0.37
Pde6a	0.00	-1.16	-0.16
Snord22	0.00	-1.16	-0.16
Tgfa	0.00	-1.16	-0.16
Ccdc42	0.00	-1.16	0.00
Treml2	0.00	-1.16	0.00
Spag6l	0.00	-1.16	0.07
9530036O11Rik	0.00	-1.16	0.07
Gm17769	0.00	-1.16	0.13
Eid3	0.00	-1.16	0.13
4930426D05Rik	0.00	-1.16	0.17
Col4a3	0.00	-1.16	0.24

Gm13710	0.00	-1.16	0.24
Gm2011	0.00	-1.16	0.24
2610305D13Rik	0.00	-1.16	0.24
4930427A07Rik	0.00	-1.16	0.63
1110017D15Rik	0.00	-1.13	-0.60
Xirp2	0.00	-1.13	-0.63
Dapl1	0.00	-1.12	-0.52
Zfp57	0.00	-1.11	-0.32
Atp10a	0.00	-1.08	-0.92
Cd300a	0.00	-1.08	-0.42
Rasal3	0.00	-1.08	-0.42
Ascc2	0.00	-1.08	-0.08
Irak3	0.00	-1.08	0.00
Plekhs1	0.00	-1.08	0.08
Gm10421	0.00	-1.08	0.21
4930593A02Rik	0.00	-1.08	0.27
AI118078	0.00	-1.08	0.32
Gm6297	0.00	-1.07	-0.39
D030068K23Rik	0.00	-1.06	-0.03
Htr2a	0.00	-1.06	-0.69
8030443G20Rik	0.00	-1.06	-0.45
Crebl2	0.00	-1.06	-0.41
Vsig10	0.00	-1.06	0.05
2900009J06Rik	0.00	-1.04	-0.02
Dmkn	0.00	-1.04	0.25
Ccdc166	0.00	-1.03	-0.06
6720483E21Rik	0.00	-1.00	-0.71
Mstn	0.00	-1.00	-0.50
Chrnb1	0.00	-1.00	-0.50
Pdlim3	0.00	-1.00	-0.50
Kcng4	0.00	-1.00	-0.50
Alox8	0.00	-1.00	-0.43
5730405O15Rik	0.00	-1.00	-0.37
Serpina9	0.00	-1.00	-0.34
Zfp54	0.00	-1.00	-0.34
Scn10a	0.00	-1.00	-0.34
Lefty2	0.00	-1.00	-0.21
Mks1	0.00	-1.00	-0.21
Dupd1	0.00	-1.00	-0.21
Amhr2	0.00	-1.00	-0.21
Scarna2	0.00	-1.00	-0.21
4921511H03Rik	0.00	-1.00	-0.21
Art3	0.00	-1.00	-0.21
Tbx6	0.00	-1.00	-0.21
Il15	0.00	-1.00	-0.21
Thbs1	0.00	-1.00	-0.10
Snx9	0.00	-1.00	-0.05

Tmem26	0.00	-1.00	0.00
Gm17821	0.00	-1.00	0.00
Card6	0.00	-1.00	0.00
Msln	0.00	-1.00	0.00
C030016D13Rik	0.00	-1.00	0.00
Gm4925	0.00	-1.00	0.16
Myh13	0.00	-1.00	0.16
Gm11762	0.00	-1.00	0.16
Prdm11	0.00	-1.00	0.16
Zfp114	0.00	-1.00	0.16
1810024B03Rik	0.00	-1.00	0.29
Wnt2b	0.00	-1.00	0.29
Oca2	0.00	-1.00	0.29
Pgbd1	0.00	-1.00	0.34
Dpys	0.00	-1.00	0.40
Btbd16	0.00	-1.00	0.40
Trim43a	0.00	-1.00	0.95

Ref.) This table presents a list of genes whose expression levels were excessively decreased due to CCH but were subsequently up-regulated to normal levels following the administration of HHex. The data highlights the impact of HHex on gene expression modulation. For each gene, the table lists the gene symbol and log4 fold-change (Log4FC) vs. sham comparison.

Table S5. Genes overexpressed by CCH and down-regulated by HHex administration.

Gene symbol	Sham	Cont	HHT
Zfp607	0.00	1.00	-0.50
Scube2	0.00	1.00	-0.37
Brip1	0.00	1.00	0.00
4930586N03Rik	0.00	1.00	0.00
6530411M01Rik	0.00	1.00	0.00
Exd1	0.00	1.00	0.00
B230110G15Rik	0.00	1.00	0.00
Sprtn	0.00	1.00	0.00
Uba1y	0.00	1.00	0.00
Zfp369	0.00	1.00	0.10
5330426P16Rik	0.00	1.00	0.29
Aldoart2	0.00	1.00	0.50
Pkhd1l1	0.00	1.00	0.50
Lrrc34	0.00	1.00	0.50
Fabp1	0.00	1.00	0.50
Ankrd42	0.00	1.00	0.50
Lat	0.00	1.00	0.50
Itgb8	0.00	1.00	0.66
Folr1	0.00	1.00	0.66
Scarna17	0.00	1.00	0.79
Mc3r	0.00	1.00	0.79
Slc2a2	0.00	1.00	0.79
Vwa5b1	0.00	1.00	0.79
Il11	0.00	1.00	0.79
Snord116l1	0.00	1.00	0.79
Fan1	0.00	1.00	0.79
4933416M07Rik	0.00	1.00	0.79
Gm14685	0.00	1.00	0.79

Kbtbd6	0.00	1.00	0.87
Prdm6	0.00	1.00	0.90
Cd59b	0.00	1.00	0.90
Edn3	0.00	1.00	0.91
1700001O22Rik	0.00	1.02	0.74
Kcnj14	0.00	1.02	0.35
4930419G24Rik	0.00	1.03	0.29
Samsn1	0.00	1.04	0.74
Shcbp1l	0.00	1.05	0.67
F2	0.00	1.06	-0.29
Gm11681	0.00	1.06	0.50
Gm6642	0.00	1.06	0.56
Sbspon	0.00	1.07	0.88
Rbm47	0.00	1.07	0.97
Mettl24	0.00	1.08	-0.50
Slc22a13b-ps	0.00	1.08	0.50
Ccdc163	0.00	1.10	0.86
Egf	0.00	1.11	0.00
Pappa	0.00	1.11	0.50
9330175M20Rik	0.00	1.12	0.45
Capsl	0.00	1.13	0.57
Cyb5rl	0.00	1.13	0.79
Lpcat1	0.00	1.16	-0.50
Trim15	0.00	1.16	0.00
Artn	0.00	1.16	0.00
Agbl1	0.00	1.16	0.00
Spx	0.00	1.16	0.29
Gm10389	0.00	1.16	0.37
Smtnl2	0.00	1.16	0.50
9030204H09Rik	0.00	1.16	0.50
Nyx	0.00	1.16	0.50
Tekt1	0.00	1.16	0.71
Lemd1	0.00	1.16	0.79
1500015L24Rik	0.00	1.16	0.79
Actrt3	0.00	1.16	0.79
Zkscan7	0.00	1.16	0.79
Cntnap5b	0.00	1.16	0.94
Snai1	0.00	1.16	0.94
Alx3	0.00	1.20	0.29
Upk1b	0.00	1.21	-0.29
Rnf141	0.00	1.21	0.00
Tnfrsf10b	0.00	1.21	0.21
Rasl11a	0.00	1.21	0.21
Eva1c	0.00	1.21	0.37
Alpk1	0.00	1.21	0.71
Pcdhga5	0.00	1.23	0.00
9630028B13Rik	0.00	1.23	0.79
Fam131c	0.00	1.23	0.79
Atp8a2	0.00	1.25	0.71
Arhgef33	0.00	1.29	0.00
Lipo1	0.00	1.29	0.00
Slc17a9	0.00	1.29	0.00
Xlr4b	0.00	1.29	0.00
Msx2	0.00	1.29	0.50
Gm4951	0.00	1.29	0.50
Cyp2s1	0.00	1.29	0.63
Hcrtr1	0.00	1.29	0.79
Rnu11	0.00	1.29	0.79
Adamts18	0.00	1.29	0.79
4930578M01Rik	0.00	1.31	0.45

Gpr149	0.00	1.35	-0.50
A830019L24Rik	0.00	1.35	0.66
Il17b	0.00	1.40	-0.50
Fignl1	0.00	1.40	0.50
Treh	0.00	1.40	0.50
Prss45	0.00	1.40	0.50
Stox1	0.00	1.40	0.79
Ksr1	0.00	1.40	0.79
Ly75	0.00	1.40	0.79
Bcl3	0.00	1.40	0.79
Hhat	0.00	1.45	0.50
Sim1	0.00	1.45	0.50
Col4a6	0.00	1.45	0.79
1700011I03Rik	0.00	1.50	0.00
Oas1a	0.00	1.50	0.50
Elf4	0.00	1.50	0.79
6430531B16Rik	0.00	1.53	0.61
Fam196b	0.00	1.54	0.50
Il12rb2	0.00	1.66	0.00
Asb4	0.00	1.66	0.79
Slc9a4	0.00	1.70	0.50
Sapcd2	0.00	1.73	0.50
2810403D21Rik	0.00	1.73	0.79
Apob	0.00	1.79	0.00
Vaultrc5	0.00	1.85	0.50
Amy2a5	0.00	1.90	0.00
Xlr4a	0.00	2.40	0.79

Ref.) This table presents a list of genes whose expression levels were excessively elevated due to CCH but were subsequently down-regulated to normal levels following the administration of HHex. The data highlights the impact of HHex on gene expression modulation. For each gene, the table lists the gene symbol and log4 fold-change (Log4FC) vs. sham comparison.

Table S6. Analysis of biological process using GO terms. $p < 0.05$ was used to identify significantly enriched GO terms.

Term	Genes count	*p*-value
DNA duplex unwinding	4	1.40E-03
Response to virus	5	3.80E-03
Cellular response to interferon-beta	4	7.10E-03
DNA replication	5	7.90E-03
DNA replication initiation	3	1.10E-02
Regulation of transcription from RNA polymerase II promoter	19	1.40E-02
Cell adhesion	10	1.50E-02
Phospholipid translocation	3	1.60E-02
Sperm axoneme assembly	3	1.70E-02
Xenobiotic metabolic process	4	2.30E-02
Multicellular organism development	14	2.30E-02
Phospholipid transport	3	2.80E-02
Negative regulation of viral process	2	3.20E-02
Flagellated sperm motility	4	3.80E-02
Triglyceride mobilization	2	3.80E-02
Organic acid metabolic process	3	4.20E-02
Exogenous drug catabolic process	3	4.40E-02

Table S7. Analysis of cellular components using GO terms. $p < 0.05$ was used to identify significantly enriched GO terms.

Term	Genes count	*p*-value
Motile cilium	6	6.70E-03
Membrane	59	8.70E-03
Extracellular region	21	1.90E-02

Table S8. Analysis of molecular function GO terms. $p < 0.05$ was used to identify significantly enriched GO terms.

Term	Genes count	*p*-value
DNA replication origin binding	4	3.80E-04
RNA polymerase II transcription factor activity, sequence-specific DNA binding	18	9.30E-04
RNA polymerase II regulatory region sequence-specific DNA binding	10	1.80E-03
Sequence-specific double-stranded DNA binding	10	9.60E-03
Oxidoreductase activity, acting on paired donors, with incorporation or reduction of molecular oxygen	4	1.90E-02
RNA polymerase II core promoter proximal region sequence-specific DNA binding	15	2.10E-02
Peptide binding	4	2.80E-02
Aromatase activity	3	3.10E-02
Single-stranded DNA binding	4	3.60E-02
Monooxygenase activity	4	4.00E-02

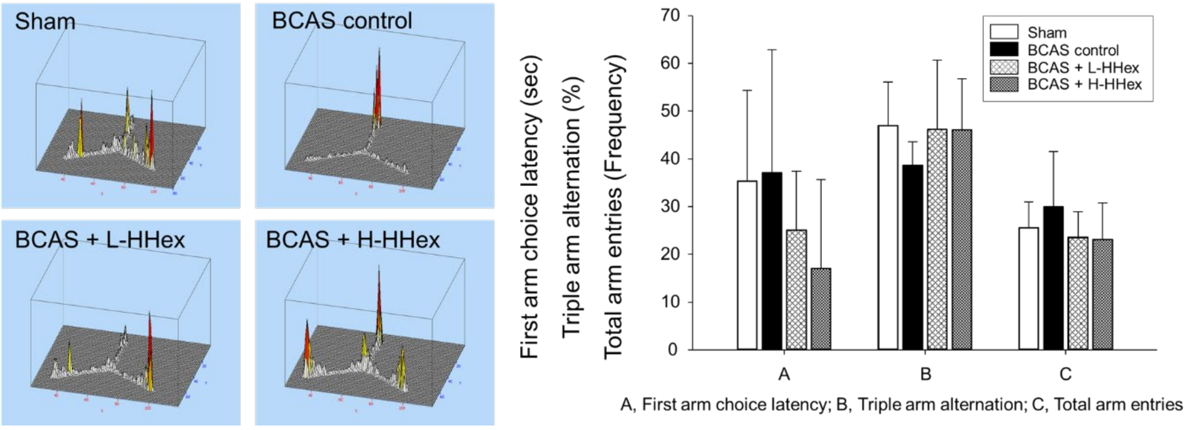

Figure S1. Results of the Y-maze behavioral test. HHex post-treatment resulted in no significant changes in first-choice latency, triple-arm alternation, or total arm entries.
